# Comparison of hyperspectral imaging and fluorescence angiography for the determination of the transection margin in colorectal resections—a comparative study

**DOI:** 10.1007/s00384-020-03755-z

**Published:** 2020-09-23

**Authors:** Boris Jansen-Winkeln, Isabell Germann, Hannes Köhler, Matthias Mehdorn, Marianne Maktabi, Robert Sucher, Manuel Barberio, Claire Chalopin, Michele Diana, Yusef Moulla, Ines Gockel

**Affiliations:** 1 Department of Visceral, Transplant, Thoracic and Vascular Surgery, University Hospital Leipzig, Liebigstr. 20, 04103 Leipzig, Germany; 2 Innovation Center Computer Assisted Surgery (ICCAS), University of Leipzig, Leipzig, Germany; 3 Institute of Image-Guided Surgery (IHU), IRCAD, Strasbourg, France

**Keywords:** Hyperspectral imaging (HSI), Fluorescence angiography (FA), Indocyanine green (ICG), Anastomotic leak, Colorectal resection

## Abstract

**Purpose:**

One relevant aspect for anastomotic leakage in colorectal surgery is blood perfusion of both ends of the anastomosis. The clinical evaluation of this issue is limited, but new methods like fluorescence angiography with indocyanine green or non-invasive and contactless hyperspectral imaging have evolved as objective parameters for perfusion evaluation.

**Methods:**

In this prospective, non-randomized, open-label and two-arm study, fluorescence angiography and hyperspectral imaging were compared in 32 consecutive patients with each other and with the clinical assessment by the surgeon. After preparation of the bowel and determination of the surgical resection line, the tissue was evaluated with hyperspectral imaging for 5 min before and after cutting the marginal artery and assessed by 6 hyperspectral pictures followed by fluorescence angiography with indocyanine green.

**Results:**

In 30 of 32 patients, the image data could be evaluated and compared. Both methods provided a comparable borderline between well-perfused and poorly perfused tissue (*p* = 0.704). In 15 cases, the surgical resection line was shifted to the central position due to the imaging. The border zone was sharper in fluorescence angiography and best assessed 31 s after injection. With hyperspectral imaging, the border zone was visualized wider and with more differences between proximal and distal border.

**Conclusion:**

Hyperspectral imaging and fluorescence angiography provide similar results in determining the perfusion border. Both methods allow a good and safe visualization of the blood perfusion at the central resection margin to create a well-perfused anastomosis.

**Trial registration:**

This study was registered at Clinicaltrials.gov (NCT04226781) on January 13, 2020.

## Introduction

Anastomotic leakage represents a serious complication after colorectal resections associated with reported rates ranging from 7 to 19.2 % [1–3]. The reasons are multifactorial, and surgeons are not able to influence all of them, for example, patient-related factors, such as age, gender, or comorbidities [4–11]. However, the surgical technique and insufficient perfusion can be determined and actively changed [7, 12, 13]. Adequate bowel perfusion is a major requirement for good anastomotic healing [14, 15]. The intraoperative evaluation of blood supply is mostly based on the surgeon’s subjective perception, including the assessment of serosal color, palpable pulsation, and signs of active bleeding from marginal arteries [13, 16, 17]. However, in order to prevent anastomotic leaks owing to poor blood circulation, there is a need to control this subjective perception by objective data. Hyperspectral imaging (HSI) is a relatively new established intraoperative imaging method, which enables the objective measurement of physiological tissue parameters. Measurements are characterized by their noninvasiveness and their freedom of contact and contrast agents [18]. HSI has already been applied in a wide medical spectrum, including the assessment of tissue perfusion, tissue classification, wound healing, or the detection of cancer [19]. In contrast, fluorescence angiography (FA) with indocyanine green (ICG) is a well-known imaging technique. Initially, it was developed for cardiological and liver diagnostics and further applied in ophthalmology [20–22]. Currently, it represents a promising method to visually assess blood flow intraoperatively and to provide a real-time presentation of perfusion of gastrointestinal anastomoses, especially of colorectal ones [22]. Both techniques, HSI and FA, offer the possibility of an intraoperative and objective control of tissue perfusion that may change the planned surgical procedure with the potential to reduce anastomotic leaks. In this study, we investigated, whether there were differences between the two techniques in determining the visualized borderline between high and low perfused tissue. Furthermore, we aimed to determine, which were the differences in localization and in time course.

## Materials and methods

### Study population

This clinical trial was implemented as a prospective, non-randomized, open-label, and two-arm study at the University Hospital Leipzig, Leipzig, Germany. Approved by the local ethics’ committee of the medical faculty of the University of Leipzig (026/18-ek), the study was registered at Clinicaltrials.gov (NCT04226781). Written informed consent was obtained from all involved patients. During the period from December 2019 to April 2020, we included 32 patients who underwent colorectal resections. These revealed right hemicolectomy, sigmoid or rectal resections. Exclusion criteria were pregnancy, nursing mothers, age under 18 years, patients who were unable to consent, and those with known hypersensitivity to ICG, sodium iodine, or iodine. These criteria were checked preoperatively by the surgeon and no potential study participants had to be excluded.

Eligible for this study were 32 consecutive patients undergoing colorectal resections. The preoperative findings from these patients are shown in Table 1. The cohort (*n* = 21 males; *n* = 11 females) had a median age of 59.5 years (range 28 to 81). Preoperative diagnosis included carcinoma (*n* = 20; 62.5 %), diverticulitis (*n* = 10; 31.3 %), adenoma (*n* = 1; 3.1 %), and Crohn’s disease with enteric fistula (*n* = 1; 3.1 %). Thirty (93.8 %) operations were performed laparoscopically and 2 (6.3 %) were open surgeries (one sigmoid cancer recurrence and one patient with several previous operations). Participants were followed up postoperatively until their discharge. Postoperative complications according to Clavien and Dindo class III or higher [23] or the occurrence of anastomotic leakage, which was defined as any disorder of the continuity of the anastomosis as detected by direct clinical signs, radiologic or endoscopic findings, were recorded [24].

**Table 1:** Preoperative findings, *N* = 32 patients

Variables	*N* (%)	Median (range)
Age in years	-	59.50 (28–81)
Sex		-
Males	21 (65.6)	
Females	11 (34.4)	
BMI	-	29 (18–40)
ASA classification		
Grade I	0 (0)	-
Grade II	26 (81.3)	-
Grade III	6 (18.8)	-
Grade IV	0 (0)	-
Previous abdominal surgeries	18 (56.3)	-
Diagnosis		
Carcinoma	20 (62.5)	-
Diverticulitis	10 (31.3)	-
Crohn’s disease	1 (3.1)	
Adenoma of the colon	1 (3.1)	
Comorbidities		
Arterial hypertonia	16 (50)	-
Hearth failure, CHD	4 (12.5)	-
Renal insufficiency	1 (3.1)	-
Diabetes type II	5 (15.6)	-
Respiratory disease	3 (9.4)	-
Liver disease	5 (15.6)	-
Alcohol abuse	11 (34.4)	-
Nicotine abuse	8 (25)	
Medication		
Blood pressure medicine	16 (50)	-
Proton pump inhibitors	9 (28.1)	-
Blood diluter	3 (9.4)	-
Oral antidiabetic	4 (12.5)	-
Statins	5 (15.6)	-
Hormones	4 (12.5)	-
Neoadjuvant therapy, *N* = 11		
Chemotherapy	10 (31.3)	-
Radiochemotherapy	7 (21.9)	-

### Hyperspectral imaging (HSI)

HSI data were acquired with the TIVITA® Tissue system (Diaspective Vision GmbH, Am Salzhaff, Germany). Measurements were performed under standardized conditions (according to our internal SOP’s = standard operational procedures) with ambient light turned off and a distance of 50 cm between object and HSI camera. By combining two dimensional-spatial data with a third spectral dimension, the system generated three-dimensional data called “hypercube.” Under illumination of the interested tissue with light in the visible and near-infrared spectrum range from 500 to 1000 nm and an acquisition time of 10 s, the analysis software provides a color and four false-color images with an effective number of 640 × 480 pixels. At a 50-cm distance, a field of view (FOV) of 8 × 6.5 cm^2^ and a theoretical spatial resolution of 0.13 mm/pixel are achieved. Furthermore, the spatial resolution was evaluated with the 1951 US Air Force (USAF) resolution test chart. At 630 nm, this resulted in an actual spatial resolution of 0.39 mm/pixel. Each false-color image represents a physiologic tissue parameter, visualized in image intensities. Tissue oxygenation (StO_2_) and near-infrared perfusion index (NIR PI) refer to the relative oxygenation of blood in the tissue. While StO_2_ relates to the oxygenation in penetration depths of about 1 mm and values ranging from 0 to 100 %, NIR PI indicates the oxygenation of structures in up to 4–6-mm depth. The distribution of water and hemoglobin content in the recorded area is visualized with the tissue water index (TWI) and organ hemoglobin index (OHI), respectively. The NIR PI, TWI, and OHI are specified in arbitrary units in the range from 0 to 100. The mentioned parameters and their determinations have already been described in detail by Holmer et al. [25].

### Fluorescence angiography (FA)

For FA, we used the VisionSense-3 iridium camera system (Medtronic GmbH, Meerbusch, Germany). The camera can be used in open and laparoscopic surgery. The (diode) laser is able to illuminate tissue with 805-nm wavelength and the sensor to detect fluorescence emission ranging from 825 to 850 nm. The level of fluorescence is visualized in relative intensities on the display with a resolution of 1920 × 1080 pixels and is measurable at any point in the image. The fluorescence intensity is measured for each pixel and ranges between 0 and 255 (8 bit coded). The system provides several real-time video images simultaneously including white light image, infrared (IR) image, and three color-fused images. The color-fused images offer augmented reality and result from overlaying IR images and white light images. A quantitative measurement in relation to the maximum value of IR intensities is possible [26, 27]. As part of this study, the measurements were performed during mini-laparotomy while extracting the specimen with a distance of 50 cm between the object and the camera. This setup lead to a FOV of 23.5 × 17.5 cm^2^ and a theoretical spatial resolution of 0.16 mm/pixel. The evaluation with the 1951 USAF resolution test chart resulted in an actual spatial resolution of 0.35 mm/pixel. To avoid possible disturbances, the light remained off during FA.

### Indocyanine green (ICG)

ICG is a nontoxic, nonionizing, water-soluble dye approved by the United States Food and Drug Administration (FDA) for human use. After intravenous or intraarterial application, most of the dye binds to plasma proteins and distributes homogeneously in the vascular system. When ICG molecules are exposed to near-infrared light with a wavelength of around 800 nm, they begin to fluoresce with a maximum wavelength of 830 nm. This emitted radiation can be recorded by an IR camera. With physiologic liver function, the dye is cleared into bile after a short plasma half-life of 3–5 min only. ICG contains sodium iodine, so allergic reactions can potentially occur. If there were any known allergies against iodine or sodium iodine, the dye was naturally not used [2, 28–31].

### Surgical technique

The standard procedure in colorectal surgery performed in our clinic has been described before by our group [24]. To assess perfusion of colonic tissue, HSI and ICG measurements were carried out before the specimen was resected and the anastomosis done. Thus, it was possible to change the site of the central resection margin, if measurements showed insufficient perfusion. After the specimens were extracted through the Alexis ring (Applied Medical, Düsseldorf, Germany) around the mini-laparotomy, the surgeon decided, where the margin of resection would be ideally placed, based on the subjective assessment of bowel serosa and the existence of pulsation of vessels. The planned proximal transection line is marked with an instrument. To measure possible deviations between the subjective evaluation of the surgeon and the objective data provided by HSI and ICG measurements, a ruler was placed alongside bowel’s longitudinal axis. After the hyperspectral camera was positioned 50 cm above the region of interest (ROI) and the light was turned off, the first HSI record was started before devascularization. In the publication by Jansen-Winkeln et al. [17], our group has already described this specific measurement in detail. Afterwards the light was turned on and the surgeon divided the marginal artery. As a part of the “cold steel” test, which symbolizes the open division of the marginal arcade, he/she judged the appearance of active bleeding as another subjective sign for adequate perfusion of the future anastomotic site. Again, the surgeon marked and potentially corrected the future transection line with an instrument. Sixty seconds after devascularization, data recording was continued as described above. According to our protocol, hyperspectral images were taken each minute for five minutes. In the following, neither the position of the instrument nor the position of the ruler was changed. The light also remained off. Only the devices were changed: TIVITA HSI camera against the VisionSense FA system. The distance of 50 cm between the camera and the object was also maintained. ICG was injected by the anesthesiologist as a bolus of 2.5 mg intravenously via peripheral access, and 10 ml saline solution was applied afterwards to make sure that the dye distributed quickly. At this time, fluorescence videography was started for 5 min. Thus, the flooding time of ICG could be determined, because the time of injection corresponded to the start of the video. Furthermore, the spread of the dye over time could be visualized. Both pictured borderlines, obtained from HSI and FA via ICG, were compared. Possible deviations to the planned transection line were recorded, and the number of changes due to perfusion assessment was documented. Deviations above 5 mm were considered relevant. The instrument for marking (scissors) is 3-mm wide, and in case of sometimes limited visibility, we have estimated 5 mm as measuring tolerance. After the operation, the hyperspectral data were analyzed with the TIVITA® Suite software and the FA imaging data with the help of ImageJ version 1.52 (Wayne Rasband, National Institutes of Health, Bethesda, Maryland, USA). For postoperative evaluation, six snapshots of the fluorescence videography were extracted via VisionSense player software: the first one after maximal initial flooding of the dye in the ROI and five more each minute after injection. Based on the previous results by our group, we decided to analyze hyperspectral data recorded 3 min after devascularization, because at this time, the biggest drop of StO_2_ and NIR PI is present [24]. Oxygenation (StO_2_) 3 min after devascularization (0–100%) was compared with the ICG data corresponding to the maximum initial flooding of the dye. Due to the existence of different measuring scales, the maximum and minimum values of each HSI and ICG image were determined. The borderline between good proximal and poor distal perfusion was defined as a 50% change. The most proximal and distal points were marked. After measuring the distances to the marking instrument, deviations between the surgically planned transection line and the measured borderlines were identified. These deviations were defined as minus (-), when they were located distally to the forceps, and as plus (+), when they were situated proximally. Moreover, the localization and the course of the borderline in HSI and ICG data were compared. In addition, to draw attention to the diffusion of the dye over time, 5 min after injection, ICG data were analyzed to show persistent fluorescence signals and their localizations.

### Statistical analysis

Data obtained from measurements and patient data were inserted into Microsoft Excel Version 16.0 (Microsoft Corporation, Washington, USA) and transferred into IBM SPSS Statistics Standard v24 (IBM Corporation, Chicago, USA) for statistical analysis (mean, median, range, standard deviation). The program was also used to create the figures, to test for normal distribution (Kolmogorov-Smirnov test) and to calculate correlations (Pearson test) and significances (Wilcoxon, *T* test). A *p* value of < 0.05 was determined as statistically significant.

## Results

In all 32 patients undergoing colorectal resections, we were able to perform HSI and ICG measurements. Nevertheless, we had to exclude two cases from our analysis. In the first case, a device failure interrupted the HSI measurement. The following ICG measurement was performed without problems. In the second case, there was no identifiable visible borderline in HSI, while the recorded fluorescence videography showed a clear border between good and insufficient perfusion. Finally, 30 patients qualified for our analysis. The information concerning the intraoperative situation with circulation-regulating drugs is shown in Table 2. All patients had an uneventful intraoperative course without relevant use of catecholamines or other perfusion-modifying drugs. Eight patients undergoing a total mesorectal excision received a protective stoma.

**Table 2:** Intraoperative findings, *N* = 30 patients

Variables	*N* (%)	Median (range)
Operation duration (in minutes)	-	256.50 (124–737)
Catecholamines		
Arterenol (μg/kg/min) *	-	0.0438 (0.0061–0.2740)
Blood pressure*		
Systolic pressure (mmHg)	-	115 (100–140)
Diastolic pressure (mmHg)	-	65 (45–90)
Oxygen saturation SaO_2_ in % * (95–99%)	-	98 (92–99)
Oxygen partial pressure pO_2_ in % * (72–107%)	-	143 (40.3–243)
Inspiratory oxygen fraction in % *	-	40 (33–59)
Blood loss (in ml) †	-	400 (0–9859)
Erythrocyte concentrate administration †	2 (6.7)	-

* at the beginning of the measurement; † over the whole operation

**Fig. 1:**
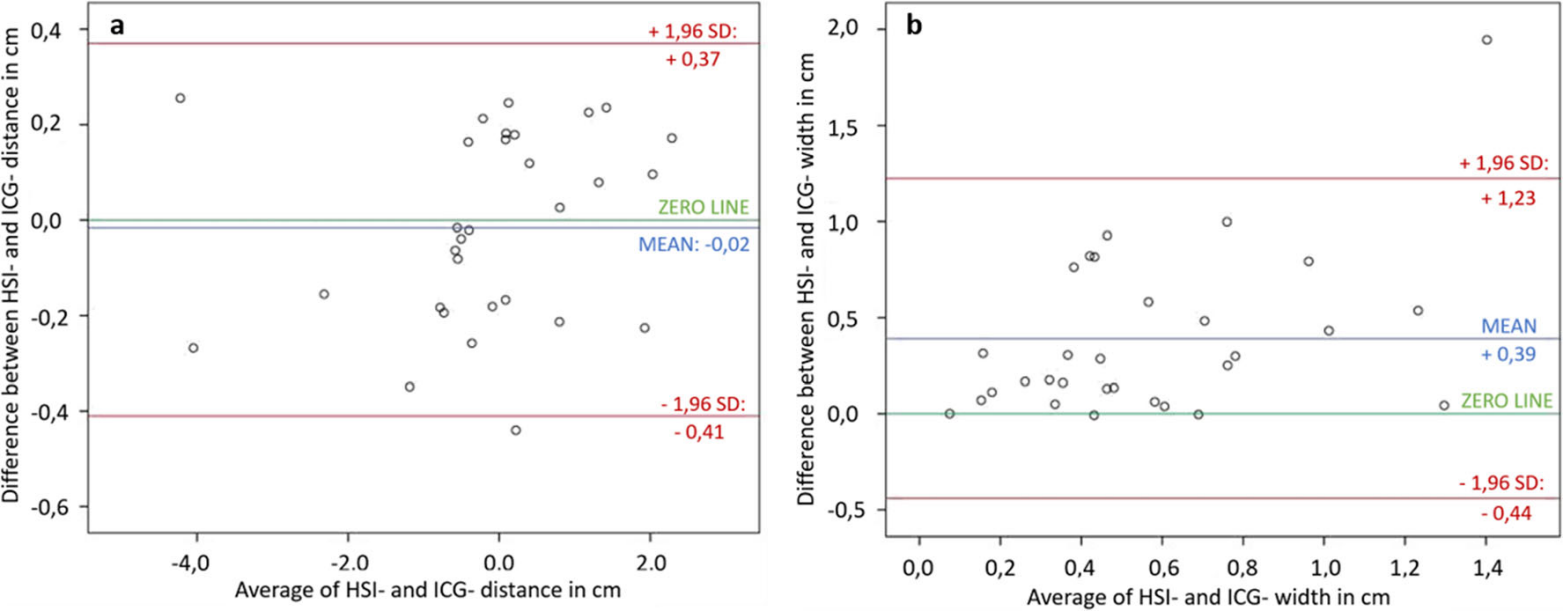
**a** Bland Altman plot of the measured distances in cm to the forceps in HSI and ICG. Scattering of the points around the zero line indicated agreement of both measured distances. **b** Bland Altman plot of the measured width in centimeters. Scattering of the points above the zero line suggested disagreement of the width of the border zone in HSI-and ICG. (SD = standard deviation)

**Fig. 2:**
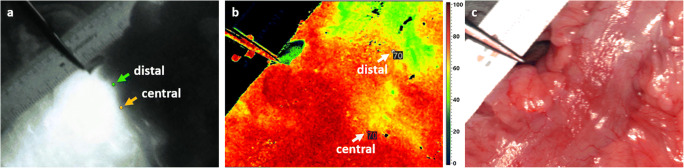
**a–c** An instrument marked the planned transection line. The ruler is best seen in the RGB picture (**c**). **a, b** Based on the respective minimum and maximum intensity of each image, border limit values were calculated. In this case, the HSI StO_2_ limit value 3 min after devascularisation was 70 and the FA/ICG value at the maximum initial flooding of the dye amounted to 131. The most central limit point in FA/ICG (**a**) and HSI (**b**) was marked and deviations to the instrument were measured. While FA pictures hardly any differences between the most proximal and distal point of the borderline (**a**), HSI shows a large distance between both points (**b**)

**Fig. 3:**
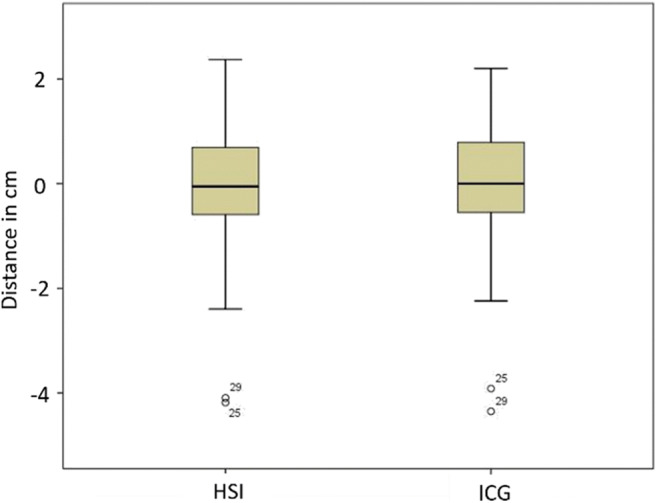
Distribution of the distance from the planned transection line to the visualized borderline in HSI and ICG data in cemtimeters

**Fig. 4:**
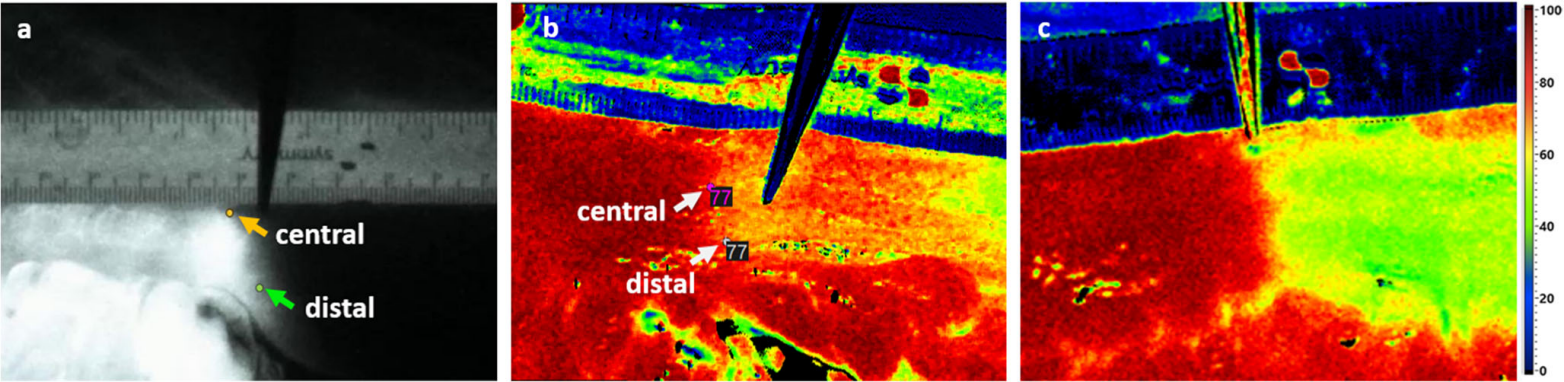
Most central and distal limit value in FA with ICG (**a**) and HSI StO_2_ (**b**). A similar course and width of both border zones are noticeable. **b** HSI StO_2_ image of a border zone with many color graduations between good proximal and poor distal perfusion. **c** Another patient’s HSI StO_2_ image with a clear and sharp borderline is showing the variety of border zones revealed with HSI

In 10 cases (33.33 %), the planned resection line was more than 5 mm distal to the demarked border zone and had to be moved proximally. The distance ranges between 0.18 and 4.35 cm with a median of 0.59 cm between the planned and the measured borderline obtained from HSI and ICG data. There was no difference between the two methods to decide upon displacing the resection line. In 12 cases, the HSI border zone was more distant from the instrument and in 3 cases the ICG border zone. In each case, the more distally located border was decisive. When considering the measured distances between the visualized borderline and the clinically planned resection line in the HSI and ICG group, the Kolmogorov-Smirnov test indicated a non-normal distribution and the Pearson’s test showed correlation between both measured distances (*R* = 0.991; *p* < 0.001). The Wilcoxon test revealed no significant differences in comparing the two mentioned distances (*p* = 0.704) and the created Bland Altman plot (Fig. 1a) with a scattering of the points around the zero line and the small bias of 0.2 (the small average of the differences) also indicated agreement of the plotted distances to the instrument in HSI and ICG data. In Fig. 2, an example of the location of the central border in ICG maximum initial flooding of the dye (a) and HSI-StO_2_ 3 min after devascularization (b) is shown. The measurements of the most central and most distal part of the border zone in ICG and HSI are also displayed in Fig. 2a and b. The distance between the planned transection line (instrument) and the pictured borderlines is presented in Fig. 3. In most of the cases, there were large differences in the width and the course of the HSI and the ICG border zone, as shown exemplarily in Fig.2. However, some measurements showed almost identical zones (Fig. 4a, b). The width of the HSI border, measured from its most proximal to its most distal point, showed a median distance of 0.66 cm (range 0.08–2.38 cm). In ICG data, this zone had a median length of 0.31 cm (range 0–1.28 cm) only. The Kolmogorov-Smirnov test demonstrated a normal distribution (width HSI *p* = 0.077; width ICG *p* = 0.20) and the Pearson analysis showed a positive correlation of *R* = 0.479 (*p* = 0.007) between the width of the measured border zones in cm in HSI and ICG data. Furthermore, the *t* test revealed significant differences (*p* < 0.001) and the constructed Bland Altman plot, with a scattering above the zero line, indicated also differences between the width of the border zone in HSI and ICG (Fig. 1b). No intraoperative complications or incompatibilities associated with ICG injection were recorded. The median time between administration and the maximum initial flooding of the ICG in the ROI was 31 s (range 19 to 47 s). After 5 min, the fluorescence signal could be detected in median 4.99 cm (range 0.54 to 8.31 cm) from the border zone that had been defined at the beginning. The median time of 12 min (range 11 to 19 min) was needed to perform both measurements. Additionally, about 30 s had to be added for the exchange of the devices.

The postoperative findings can be found in Table 3. Postoperative anastomotic leakage occurred in one patient undergoing a primary robotic-assisted rectal resection in rectal cancer without neoadjuvant therapy (*n* = 1; 3.33 %). The intraoperative HSI and FA visualized a well-perfused tissue at the planned transection line and no intraoperative complications were recorded in this case. On the seventh postoperative day, the routine control rectoscopy showed a small dehiscence of the anorectal anastomosis. The leakage consolidated conservatively under “endosponge” therapy (BBraun, Melsungen, Germany) within 16 days. Afterwards this patient had an upper gastrointestinal bleeding due to an undetected gastric carcinoma. The further postoperative cause was uncomplicated and wound healing proceeded per primam. Robotic gastrectomy was carried out in this patient timely. In addition to this case, postoperative complications according to Clavien-Dindo class III and higher were observed in one more case (*n* = 2; 6.67 %) with ureter leakage after open surgery for recurrent sigmoid carcinoma.

**Table 3:** Postoperative findings (*N* = 30) and tumor characteristics in the related group (*N* = 19)

Variables	*N* (%)	Median (range)
Hospital stay in days	-	9 (5–25)
Anastomotic leakage	1 (3.3)	-
Clavien-Dindo classification		
Grades III–V	2 (6.7)	-
Tumor characteristics		
UICC classification		
Stage 0 (incl. yp0)	2 (3.3)	-
Stage I	3 (6.7)	-
Stage II	6 (20.0)	-
Stage III	7 (23.3)	-
Stage IV	1 (3.3)	-

## Discussion

Anastomotic leaks after colorectal resection are not only associated with severe short-term consequences, such as an extended hospital stay or the need for reoperations, but also with the risk of suffering from long-term deteriorations, like diminished quality of life and cancer recurrence. Thus, anastomotic leakage contributes to an increase in morbidity and mortality [7, 15]. An approach to reduce the incidence rate and its consequences represents the identification of patient-related risk factors and their reduction. However, these factors are often beyond our control. Another important aspect to prevent leaks is the impact of the performed techniques and the existence of adequate perfusion. Many methods are known to evaluate intestinal perfusion intraoperatively, but due to technically difficulty of handling, expensive equipment, or an inadequate reproducibility, some of them have not been integrated into the daily clinical routine [13].

In this clinical trial, we compared two evolving methods to visually assess perfusion, namely HSI and FA via ICG, in exactly the same patients. This is, to our knowledge, the first study of this kind with this objective. In 30 cases, a successful imaging was possible by both methods, which led to a change in the surgical strategy in 10 cases. While the width of the two compared border zones differed significantly, we were not able to detect a significant difference between the clinically relevant, most proximal border points. Including all cases, we recorded a leakage rate of 3.3 % (*N* = 1).

Concerning the change of the surgical procedure, we demonstrated that in 33 % of all cases, the transection line, as selected by the surgeon’s subjective assessment, could be corrected from a too far distal position to a better perfused proximal area. Furthermore, the differences between the subjective assessment and the objective data set are an indicator for inaccuracies of a purely subjective assessment. In the PILLAR II trial, Jafari et al. described a change in the surgical strategy due to perfusion imaging via ICG in 7.9 % of the patients [2]. Jun Watanabe and colleagues reported a similar number of 5.7% cases, in which FA led to a change of the proximal transection line [32]. A previous study of our group had listed a change from a too far distal to a better perfused proximal area in 13 cases by means of HSI imaging performed during 20 colorectal resections [24]. Our current study clearly demonstrates that both imaging methods, HSI and ICG, can optimally visualize the borderline between good proximal and poor distal perfusion and that there are no significant, clinically relevant differences in the location of the most proximal border point. In contrast, the width of the border zone—measured at the most proximal and most distal points of the border zone (Fig. 2a + b)—differs significantly between HSI and ICG: 0.66 cm in median for the HSI and 0.31 cm for the ICG measurement. While the borderline in ICG images can be described as clear, sharp, and straight, the ones by HSI seem to run more inconsistently and offer more nuances in most of the cases. An example of both variations is shown in Fig. 4b and c**.** We were not able to find an explanation or a clinical context for the various phenomena of the visualized border zone in hyperspectral images. On the one hand, hyperspectral images with a long-distance border (like the one in Fig. 4b) seem to provide more detailed information about perfusion, but on the other hand, this may lead to problems in identifying the exact course of the borderline. At this point, clear cutoff values, which differentiate precisely between good and insufficient perfusion, are urgently needed. Currently, no exact values, that must be reached to avoid the occurrence of anastomotic leaks in colorectal surgery, are known. To define these values, further studies are ongoing by our group and others.

In this study, two cases had to be excluded due to insufficient HSI data. These cases show that the use of FA with ICG is easy. Referring to Wada et al., the median time from ICG administration to the appearance of a fluorescence signal was 39 s [13]. Ris and colleagues reported a median time of 29 s until fluorescence detection [13, 29]. In this study, we found a median time of 31 s (range 19–47 s) between ICG injection and the maximum initial flooding of the dye. We were not able to prove a correlation between the flooding time and the occurrence of anastomotic leaks. However, it is of high clinical relevance to evaluate the visualized borderline according to its localization and course directly after the appearance of the first visible maximum fluorescence signal. In this study, we noticed a shift of the borderline with a median distance of 4.99 cm (range 0.54 to 8.31 cm) 5 min after injection. Due to the capillary flow diffusion of the dye over time, the visible fluorescence signal may reach too far distal ischemic areas. Therefore, the risk of overestimating the actual well-perfused tissue zone increases [33]. As a result of this, anastomotic leakage based on poor blood supply may increase.

In this clinical trial, both imaging methods proved to be feasible, simple, and reliable with minimal differences according to measurement inaccuracies. While HSI is known as a noninvasive and contactless imaging method [18], the literature describes a small percentage of allergic complications caused by ICG administration [31]. In our current study, there were no complications due to the injected agent and the procedure of FA turned out to be safe. Nevertheless, in cases of known hypersensibilities, the agent should be used with caution [28, 34]. In addition, with a median time of 31 s until visible fluorescence signal, it is a rapid possibility to assess perfusion, which hardly changes the duration of surgery. In HSI, however, 3 min after devascularization, the biggest drop of perfusion is noted [24]. Thus, it is important to evaluate data after 3 min and consequently, it takes longer time than FA via ICG. But in contrast to fluorescence imaging, HSI can be performed repetitively with no imaging artifacts of previously injected dye. Due to the distribution of ICG in blood flow and a plasma half live ranging from 3 to 5 min [28, 34], the procedure cannot be repeated within short time intervals. In all 30 cases, the IR camera detected fluorescence radiation even 5 min after injection. This indicates the long presence of ICG molecules in the circulation. Therefore, it is necessary to ensure that no ICG fluorescence is present in the tissue before performing another ICG measurement. Repeated ICG applications to evaluate perfusion should be considered very critically. In addition, the ICG costs are not insignificant and should be considered using FA.

Several limitations are related to this study. Firstly, the small sample size might be a point of criticism. Studies with more participants and a control group are needed to clarify the influence of HSI and fluorescence imaging on the occurrence of anastomotic leakage. Beyond that, our investigations considered the proximal site of the future anastomosis only. In addition, the distal part and the finished anastomosis should be included, too. With regard to FA, a bolus of 2.5 mg ICG was injected in our study. By a dosage adapted to the individual body weight, higher reproducibility and accuracy could be achieved. For example, Watanabe et al. used a dose of 0.25 mg/kg body weight for FA via ICG in laparoscopic low anterior resection and in all cases a high contrast fluorescence signal was obtained [32]. However, according to the literature, the administration of ICG in the form of a bolus is widely used. For example, Jafari et al. described in the PILLAR (II) trial the injection of an ICG bolus ranging from 3.75 to 7.5 mg for FA [2] and Kawada et al. administered a bolus of 5 mg for FA in laparoscopic colorectal surgery [12]. The use of the ideal dose may be complicated by the lack of an established standardized dose [12]. Additionally, a problem of all these studies is that only the anterior side of the bowel, usually less than 1/3 of the circumference, can be assessed. The mesenteric side is considered to be better perfused, but visualization is only possible in a part of the intestinal circumference. This results in a diagnostic gap in the assessment of intestinal perfusion.

Finally, this study demonstrated that hyperspectral and FA are well comparable. With regard to the definition of the borderline during colorectal resections, there are no clinically relevant, significant differences between the obtained HSI and ICG data. With their respective advantages and disadvantages, both procedures provide reliable and identical results referring to the dimension of resection, which is necessary to provide optimal conditions for physiological healing.

## Conclusion

Our data clearly demonstrate, that hyperspectral- and fluorescence imaging are well comparable and are complementary methods, each with its advantages and disadvantages, strengths and weaknesses. With regard to the definition of the borderline during colorectal resections, there are no clinically relevant, significant differences between the obtained HSI- and ICG-data. Both methods provide reliable and comparable results for determining the ideal resection line for optimal anastomotic healing.
