# Correction to: Comparison of hyperspectral imaging and fluorescence angiography for the determination of the transection margin in colorectal resections—a comparative study

**DOI:** 10.1007/s00384-022-04227-2

**Published:** 2022-08-02

**Authors:** Boris Jansen-Winkeln, Isabell Germann, Hannes Köhler, Matthias Mehdorn, Marianne Maktabi, Robert Sucher, Manuel Barberio, Claire Chalopin, Michele Diana, Yusef Moulla, Ines Gockel

**Affiliations:** 1 Department of Visceral, Thoracic and Vascular Surgery, University Hospital Leipzig, Liebigstr. 20, 04103 TransplantLeipzig, Germany; 2 Innovation Center Computer Assisted Surgery (ICCAS), University of Leipzig, Leipzig, Germany; 3 Institute of Image-Guided Surgery (IHU), IRCAD, Strasbourg, France

## Correction to: International Journal of Colorectal Disease (2020) 36:283–291https://doi.org/10.1007/s00384-020-03755-z

In the original published version of the above article contained missing information. The equal contribution of the authors Yusef Moulla and Ines Gockel information should have been presented as follows:

“Yusef Moulla and Ines Gockel contributed equally as last authors of this article.”

The original article has been corrected.

